# An Audit of Orthopaedic Discharge Summaries Comparing Electronic With Handwritten Summaries: A Quality Improvement Project

**DOI:** 10.7759/cureus.39396

**Published:** 2023-05-23

**Authors:** Rakshith Chakravarthy, Mohammed Shahid, Moinuddin Basha K, Sachin P Angadi, Nagesh Sherikar

**Affiliations:** 1 Orthopaedics, MVJ (MV Jayaraman) Medical College and Research Hospital, Bangalore, IND

**Keywords:** discharge summaries, quality improvement, electronic record, summaries, handwritten ds, electronic ds

## Abstract

Introduction

Discharge summaries (DS), which are sent from inpatient to outpatient settings, transmit critical clinical information. DS play a crucial role in the discharge process since they provide critical information about the patients that is simple to remember and help with patient follow-up in the community. This audit sought to determine if a quality improvement (QI) program may have an influence on the severity of mistakes at the moment of discharge and to assess the existing degree of inconsistencies on handwritten DS for orthopaedic patients.

Methodology

From the orthopaedics department at a tertiary care facility in south India, 100 handwritten DS and 100 electronic DS over six months were randomly chosen, and they were retrospectively audited against a predetermined set of criteria. The errors were compiled and compared by three reviewers.

Results

Some of the criteria, such as the doctor's signature, the speciality of admission, procedural therapy at the hospital, and the date of admission, were contained in all handwritten and electronic DS. Some of the metrics showed that electronic DS performed better than handwritten DS in areas such as hospital complications, which increased from 50% to 100%, contact information, which increased from 34% to 95%, and condition at discharge, which increased from 66% to 96%. Also, understandability increased from 58% to 100%, prognostic details increased from 70% to 96%, allergies increased from 66% to 100%, physical examination findings increased from 88% to 100%, admission diagnosis increased from 80% to 100%, patient/physician details increased from 92% to 100%, the information given to patient increased from 88% to 100%, problem list/issue pending increased from 35% to 92%, investigation increased from 80% to 100%, discharge medications increased from 88% to 100%, follow-up plan increased from 80% to 100%, discharge diagnosis increased from 94% to 100%, International Classification of Diseases, Tenth Revision (ICD-10) code increased from 93% to 100%, and days of admission increased from 92% to 100%.

Conclusion

Following the deployment of electronic DS, we were able to better care for patients and lessen their discomfort. We advise converting to electronic DS to enhance patient care and better record-keeping since this will become a significant problem if all notes are not accurately filled and are not readable.

## Introduction

There is a correlation between four out of 100 discharge summaries (DS) and rates of readmission [1,2] and adverse events that occur after release [3]. They are essential, and the Joint Commission on Accreditation of Healthcare Organizations mandates that certain components be included in each application for accreditation of a healthcare organization [4]. Discharge reports are currently not uniform across institutions, and post-discharge visits do not anticipate their availability. In several attempts to improve the quality of DS, more organized forms or computer-generated summaries have been used [5,6]. They have also produced a recurrence of major mistakes and omissions [7].

In modern medicine, an audit is used more and more frequently. While detecting, evaluating, and avoiding inpatient morbidity and mortality are given a lot of attention, guidelines are surprisingly underemphasized when it comes to making sure that DS contain accurate information for family doctors. Furthermore, accuracy is crucial since the coding data used to create computer-generated DS frequently serves as the foundation for hospital, regional, and national audit data. An essential record that details the patient's hospital stay from admission through discharge is the DS. Additionally, it offers essential data for patient follow-up in the same hospital or a different setting. Clinicians will greatly benefit from having a succinct electronic patient record that incorporates these materials in a manner that is simple to read and use, and it will also increase patient safety. Patients might suffer if adequate documentation is not done, as it is a crucial form of written communication required for the continuation of therapy.

The rapid transmission of correct and appropriate diagnostic results, treatment plans, complications, consultations, tests that are still pending release, and preparations for post-discharge follow-up can help improve the consistency of this handoff [8,9]. Delayed or inaccurate communication among healthcare providers after a patient's discharge can negatively affect the treatment continuity, patient safety, the satisfaction of both patients and clinicians, and the proper utilization of resources. Primary care physicians may not be aware of a patient's hospitalization and specific follow-up requirements, which can delay early post-discharge follow-up for complex medical issues. Dissemination of information regarding a patient's prescriptions is also a big issue in health care, and inadequate medical information transmission during transition points can lead to adverse drug events and medication mistakes in hospitals. Initiatives have been created to improve the procedure [10]. There have been a lot of different attempts made to improve the quality of DS by making use of more structured formats or computer-generated summaries. While these attempts have had some success in terms of thoroughness, clarity, and practitioner satisfaction [5,6], there is still a problem with serious errors and omissions [7], which leads to DS that are of poor quality and a lack of availability at the point of care [11].

Adopting a consistent approach to recording DS is critical to enhancing our clinical practice and ensuring that our patient records have all the information required to provide the highest quality of care. Transitions of care posed a chance for drug mistakes since our institution employed handwritten discharge statements. At this interface, communication hiccups are frequent. The discharge prescription must close this communication gap for the subsequent healthcare provider (HCP) to administer the appropriate medication in a safe and timely manner. The purpose of this study is to determine if the utilization of an electronic DS improved the accuracy of DS and reduced the number of errors overall.

## Materials and methods

The rural populace in Bangalore, India, may access tertiary-level orthopaedic care at reasonable prices because of the orthopaedic surgery unit at the hospital. This being a quality improvement (QI) project without health sciences research (HSR) elements involved, there is no requirement for independent institutional review board (IRB) approval.

At the time of release, patients receive handwritten summaries of their diagnoses, treatments, and recommendations. This gave rise to several preventable mistakes that had an impact on future follow-up and other healthcare professionals engaged in the patient's continued care. Electronically produced DS were implemented on January 8th, 2022 in our organization in consideration of all the aforementioned factors. Orthopaedic residents input data into a standardized form and the data are stored on a computer for later use. The pertinent consultant, the primary complaint, the primary diagnosis, the treatments (whether they were operational or procedural), the complications, the revisions to the medicine, and the follow-up plans are all included in the submitted data. In addition, free material is inserted in the document at the discretion of the medical personnel to give an adequate explanation of patient treatment. Between May 22 and October 22, a retrospective audit of 200 DS, of which 100 were handwritten and 100 were electronic, was carried out to determine whether or not the quality of the summaries had improved since the introduction of electronic media. The audit sample consisted of both handwritten and electronic DS. The systematic review provided a set of standard checklists that were used to evaluate these discharge reports [12].

## Results

A total of 200 discharge reports were examined using a set of criteria. The results were analysed and are summarized in Table 1 given below.

**Table 1:** Proportion of handwritten vs. electronic discharge summaries adhering to the defined criteria. DS: discharge summaries; ICD-10: International Classification of Diseases, Tenth Revision.

Parameter	Handwritten DS (%)	Electronic DS (%)
Doctor sign summary	100	100
Speciality of admission	100	100
Date of admission/discharge	100	100
Procedure treatment at the hospital	100	100
Discharge diagnosis	94	100
ICD-10 code	93	100
Patient/physician details	92	100
Days of admission	92	100
Physical examination findings	88	100
Discharge medications	88	100
Follow-up plan	80	100
Admission diagnosis	80	100
Investigations and results	80	100
Allergies	66	100
Understandability	58	100
Complications in hospital	50	100
Information given to the patient	88	97
Prognostic details	70	96
Condition at discharge	66	96
Contact information	34	95
Problem list/issues pending	35	92
Coping support	0	100
Reminder to bring the documentation next time	0	100
Optional nursing comments	0	100
Resuscitation status	0	100
Pain relief	0	100
Complementary and alternative medicine use	0	100
Nutrition	0	100
Patient sign	0	100
Social issues relevant to management	0	0
Religious/cultural concepts	0	0
Support to relatives	0	0
Palliative care information	0	0
Discharge destination	0	0
Clinical trial involvement	0	0
Sick note	0	0

This table shows the percentage of various parameters being recorded accurately in handwritten and electronic DS. Statistics are not needed for QI projects of this nature and only descriptive and exploratory analysis can be done.

Doctor sign summary, the speciality of admission, date of admission/discharge, and procedure treatment at the hospital have a 100% rate for both electronic and handwritten DS, indicating that they are well-documented.

The mention of discharge diagnosis, International Classification of Diseases, Tenth Revision (ICD 10) codes, patient/physician details, days of admission, physical examination findings, discharge medications, follow-up plan, admission diagnosis, investigations and results, allergies, understandability, and complications in hospital have a 100% rate for electronic DS, while it is lower in handwritten DS.

The information given to the patient, prognostic details, condition at discharge, contact information, and problem list/issues pending though not 100%, have a higher rate in electronic DS compared to handwritten DS.

The documentation regarding coping support, a reminder to bring documentation next time, optional nursing comments, resuscitation status, pain relief, complementary and alternate medicine use, nutrition, and patient sign have a 100% rate for electronic DS while it is 0% for handwritten DS, indicating that it is not routinely mentioned in handwritten DS.

However, the social issues relevant to management, religious/cultural concepts, support to relatives, palliative care information, discharge destination, clinical trial involvement, and sick note have a rate of 0% for both handwritten and electronic DS, indicating that they are not routinely documented.

## Discussion

In addition to playing an important role in patient care, DS also play an important role in teaching and research. As a result of an increased number of medical negligence and malpractice lawsuits, precise record-keeping of patient notes has become a necessity. In the course of this audit, one of the most important documents of general medical practice and, more specifically, surgical treatment was examined.

In addition to negatively impacting the accuracy of local and national audit data, many mistakes have significant clinical ramifications that affect patient care. They also have financial and legal ramifications for health care. The calibre of the DS influences an institute's reputation in part. Summaries of low quality and errors reflect poorly on the institution from which they were produced.

Follow-up plans were needlessly burdensome due to incorrect identification of the treatment unit and department. Poor prescription advice may be deadly, especially when it involves critical medications. It was challenging for the patient to get emergency care when necessary because there was no emergency number on the handwritten summary.

A study conducted by Callen et al. [13] showed that medication error in DS was about 10%, which was similar to the finding of discharge medications in our study. Some studies [2,14-16] have indicated that mistakes are connected with this junior status. According to a study by Wilson et al. [15], the primary diagnosis was not provided in 10.4% of DS, 18.7% of DS failed to mention the patient's presenting problem, 41.6% of DS failed to mention allergies or reactions, 64.8% of DS failed to mention results-pending information, 44.2% of DS failed to mention operations or procedures, 44.2% failed to mention complications, and 20.7% failed to mention medications on discharge, and as compared to this study, our audit reported significantly higher numbers in the handwritten DS group.

A study by Naidu et al. [17] showed that all patients (100%) received a DS and a carbon copy of the same was retained in the hospital. In our study, the same was done for handwritten DS. The three crucial elements, namely, the diagnosis, prescription, and discharge instructions, had readability scores of 66%, 76%, and 65%, respectively. In our study, the handwritten DS group's legibility score was 58%, but it increased to 100% in the electronic DS. In the prescription, the dose and duration were written in more than 90% of the cases, comparable to our research, which had a dosage and duration written in 88% of the cases and 100% of the cases in the electronic DS group. In 80% of the cases, the patient's signature was collected only after being fully informed of the discharge instructions; however, in our handwritten DS example, it was totally missing but was included afterwards in the electronic DS group. The recording of investigative findings and recommendations for action has serious flaws. A total of 80% of the handwritten DS were not documented, and 11% of the doctors' DS were not signed. In our study, all of the DS were signed by the doctors.

When case notes are poorly ordered and contain several entries, it may be difficult to find the material that is relevant to the investigation. These issues might not have occurred in the first place if the standardized form, which serves as the foundation for the summary, had been used. In addition, when confronted with a significant number of discharges, many people might be unwilling to invest the time required to locate the correct code. It may be challenging to decode codes, and handwritten codes are even more prone to mistakes.

Errors in patient details and the department that is accountable for them may be avoided if the information about patients could be retrieved from the computer system that is used for main patient administration at the hospital. A person who is familiar with the patient should be the one to write the summaries that are included in the discharges. The consultant who is in charge of the patient should draft complex summaries. Additionally, surgical residents should be instructed on how to use an electronic database and DS as well as its advantages.

Clinical trial involvement, engagement, coping support, social concerns important to management, help for the family, palliative care information, sick note, and discharge destination were the characteristics that were absent from both the handwritten and computerized DS. A few things were solely absent from the handwritten DS, such as the patient's signature, optional nurse remarks, resuscitation status, pain management, usage of complementary and alternative medicines, and allergies. From a legal, professional, and patient care continuum viewpoint, documentation has been crucial. For a very long time, governments with few resources have not given much attention to documentation. One of the most fundamental clinical tools is the patient's record, which is used in practically every consultation. To ensure that patients receive the best clinical care possible, patient records serve to paint an accurate and clear picture of their care and treatment. They are crucial to ensuring that an individual's evaluated requirements are satisfied completely and promptly because they facilitate clinicians' communication with one another, with other healthcare professionals, and with themselves. When they are all combined, they provide a permanent record of individual considerations and motives for actions. They are frequently given little priority, are poorly maintained, and are not always available while being necessary for effective communication and appropriate clinical treatment. In conclusion, electronic DS are superior to handwritten ones due to their accuracy, timeliness, accessibility, organization, legibility, and standardization.

However, there are some limitations to this study. As the study was conducted in a single centre, the results may not apply to the rest of the tertiary care hospitals that have more advanced IT services. Also, this was a retrospective review, and although steps were taken to have unbiased reviewers of the DS, this could have introduced selection bias. Another limitation was that the time taken between the DS being handwritten and typed was not measured in this audit, but this is something that could be addressed in future audits. This could help us to know if the time consumption defers the surgeon from typing the DS if they do not have proficiency in typing.

## Conclusions

The findings of the audit indicate that the orthopaedics department of our institution could improve its compliance with mandatory documentation by utilizing the electronic DS system. Our audit revealed that engaging surgeons at all levels in collaborative discussions and educational initiatives can bring about positive changes. However, if surgeons omit information from the proforma, a written discharge narrative may not provide a complete picture. Some experienced surgeons may still prefer to take notes by hand, but we recommend switching to the electronic DS system for improved patient care and accurate record-keeping. Failure to fill out all the necessary fields and legibly document information could become a significant problem. These findings are only applicable to this single institution and cannot and should not be extrapolatable to any other setting, even those similar to this single institution.
